# Alterations in Gastric Microbiota After *H. Pylori* Eradication and in Different Histological Stages of Gastric Carcinogenesis

**DOI:** 10.1038/srep44935

**Published:** 2017-03-21

**Authors:** Tung Hiu Li, Youwen Qin, Pak Chung Sham, K.S. Lau, Kent-Man Chu, Wai K. Leung

**Affiliations:** 1 Department of Medicine, The University of Hong Kong, Hong Kong; 2 Centre for Genomic Sciences, The University of Hong Kong, Hong Kong; 3 Department of Surgery, The University of Hong Kong, Hong Kong

## Abstract

The role of bacteria other than *Helicobacter pylori* (HP) in the stomach remains elusive. We characterized the gastric microbiota in individuals with different histological stages of gastric carcinogenesis and after receiving HP eradication therapy. Endoscopic gastric biopsies were obtained from subjects with HP gastritis, gastric intestinal metaplasia (IM), gastric cancer (GC) and HP negative controls. Gastric microbiota was characterized by Illumina MiSeq platform targeting the 16 S rDNA. Apart from dominant *H. pylori*, we observed other *Proteobacteria* including *Haemophilus, Serratia, Neisseria* and *Stenotrophomonas* as the major components of the human gastric microbiota. Although samples were largely converged according to the relative abundance of HP, a clear separation of GC and other samples was recovered. Whilst there was a strong inverse association between HP relative abundance and bacterial diversity, this association was weak in GC samples which tended to have lower bacterial diversity compared with other samples with similar HP levels. Eradication of HP resulted in an increase in bacterial diversity and restoration of the relative abundance of other bacteria to levels similar to individuals without HP. In conclusion, HP colonization results in alterations of gastric microbiota and reduction in bacterial diversity, which could be restored by antibiotic treatment.

Gastric cancer (GC) is the third commonest cause of cancer related death in the world^1^. In particular, more than half of GC related deaths occurred in East Asia. Although *Helicobacter pylori* (HP) has been classified as a group I carcinogen by the International Agency for Research on Cancer (IARC) more than two decades ago^2^, the exact mechanism underlying how HP induced gastric carcinogenesis remains poorly understood. Histologically, HP infection invariably leads to chronic gastritis, which progresses to atrophic gastritis (AG), intestinal metaplasia (IM), dysplasia and ultimately to cancer in some individuals^3^. Hence, AG and IM are generally considered to be premalignant gastric lesions.

Apart from HP, little is known about the role of other bacteria on the development of GC. Gastric atrophy has long been associated with bacterial overgrowth in the stomach due to the loss of gastric acidity. It has been shown that treatment with proton pump inhibitor could result in bacterial colonization of the stomach^4^. However, there are difficulties in characterizing these organisms in the stomach by traditional bacterial cultures in the past. With the availability of next generation sequencing (NGS), it is now possible to identify these previously unknown organisms in the stomach. Several studies have attempted to characterize the gastric microbiota in patients with and without HP infection as well as in patients with GC with conflicting results^5^,^6^,^7^,^8^,^9^,^10^, possibly due to the use of different platforms and in different patient’s groups. Moreover, it remains to be determined whether the disturbed gastric microbiota in HP infected subjects could be reversed by HP eradication therapy.

This study attempted to characterize the changes in gastric microbiota after HP eradication and the changes in gastric microbiota in individuals with different histological stages of gastric carcinogenesis.

## Results

### Patients’ samples

We sequenced 60 biopsy samples from 33 individuals including subjects with HP-associated chronic gastritis, gastric IM, gastric adenocarcinoma and HP-negative controls (Supplementary Table 1). A total of 13,575,770 16 S rDNA V3-V4 sequencing reads were yielded. After a series of quality control processing, 6,228,692 (45.9%) high quality non-chimera reads were retained and were clustered into 874 non-singleton operational taxonomic units (OTUs). On average, each sample had 103,812 ± 45,764 reads and 135 ± 42 OTUs (Supplementary Table 2). The rarefaction analysis suggested that the sequencing depth has the ability to recover most of the community’s diversity (Supplementary Figure 1).

**Figure 1:**
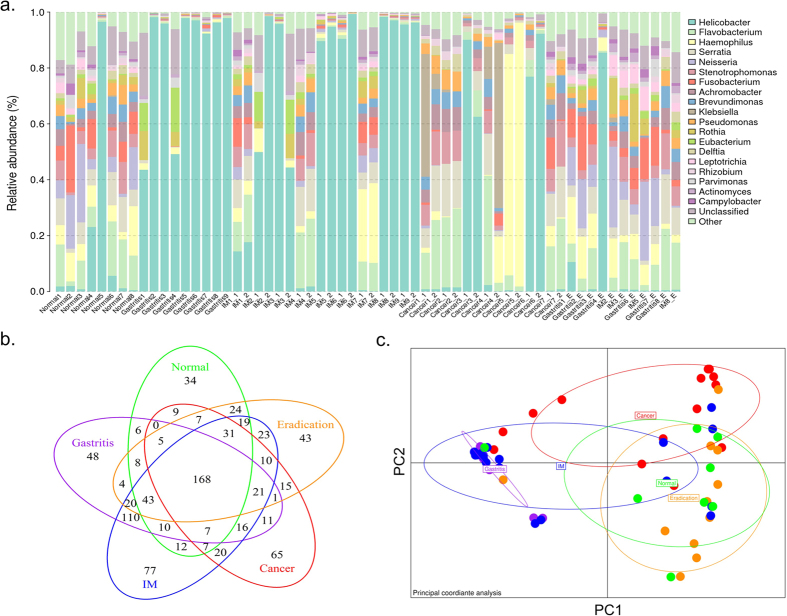
Genus composition and dispersion of 60 sequencing samples. (**a**) The relative abundance distribution of major genera across 60 sequencing samples. Only genera with relative abundance > 0.5% were shown, and the others were grouped into “Other” item. Genera were sorted by the decreasing order of average relative abundance. Samples were ordered according to their histological information. Samples with the same prefix were from the same individual. For IM samples, 1 indicates antral biopsy sample while 2 is corpus biopsy sample. For cancer samples, 1 indicates adjacent tissue biopsy sample while 2 is tumor biopsy sample. The “E” suffix represents samples after HP eradication. (**b**) The Veen plot of common OTUs among different group of samples. The number indicates the number of shared OTUs. (**c**) Principle coordinate analysis (PCoA) based on OTUs relative abundance profile. The variance explained by PC1 and PC2 are 55.3% and 13.3%, respectively. The distance was measured by root Jessen-Shannon divergence (rJSD). Points represent samples, and the color indicates group information. The accompany dendrogram was given in Supplementary Figure 5.

More than half of the reads (55.8%) were grouped into one OTU which was annotated as *H. pylori*. Together with 17 other OTUs which belonged to five phyla: *Proteobacteria* (13 OTUs), *Bacteroidetes, Firmicutes, Fusobacteria* and *Actinobacteria*, they accounted for 90.2% of the sequencing data. Moreover, these five phyla were mainly consisted of several genera (Supplementary Figure 2). Apart from these five major phyla, others were also identified in our dataset with low abundance (Supplementary Figure 3). At genus level, 19 genera were identified with average relative abundance > 0.5% (Fig. 1a). The high abundance genera were *Helicobacter, Flavobacterium, Haemophilus,* and *Serratia*.

The OTUs can be divided into several categories according to their occurrences among different sample groups (Fig. 1b). We identified 168 core OTUs which appeared in all histological groups. In total, these OTUs accounted for 99.1% (6,170,218/6,228,692) of the sequencing reads, suggesting that they are the major components of the human gastric microbiota. Most of the core OTUs (135/168 = 80.4%) can be annotated to genus level, which was higher than the overall annotation rate (552/874 = 63.2%). On the other hand, the cancer group specific OTUs, were more likely to originate from unknown species.

### Gastric Microbiota in Different Stages of Gastric Carcinogenesis

*Proteobacteria* was the dominant phylum in both HP negative and HP positive samples. While HP was the dominant bacterium in HP positive samples, other *Proteobacteria* including *Haemophilus, Serratia, Neisseria,* and *Stenotrophomonas* dominated the HP negative samples (Fig. 1a). Apart from HP, *Eubacterium* was found to be enriched in HP gastritis group. There was no significant difference in the bacterial compositions between the paired antrum and corpus samples. Notably, both the paired antrum and corpus as well as the paired tumor and adjacent non-tumor tissue samples had considerably similar bacterial compositions as shown by PERMANOVA analysis (Supplementary Table 3).

In order to show the similarity of different samples, we performed PCoA analysis based on OTUs relative abundance profiles. The 2-D plot of the first two principal coordinates shows a clear divergence of cancer samples with other samples (Fig. 1c). This pattern remained even after removing the HP related OTUs (Supplementary Figure 4). HP gastritis samples overlapped with IM samples even after excluding HP OTUs.

OTU1, which belonged to HP, had the strongest negative correlation (Spearman correlation coefficient = −0.87, P < 1e-6) with the first principal coordinate. In addition to PCoA analysis, we conducted hierarchical clustering analysis using four widely used distance metrics to avoid the bias of single distance metric and obtain consistently robust results. The dendrogram graphs show that samples were converged according to both HP and disease states (Supplementary Figure 5). While there is a clear dispersion of HP negative and HP positive samples, GC samples tended to group together. Although the effect of HP status was largely reduced by removing HP OTUs, the convergence of GC samples still remained (Supplementary Figure 6). The LEfSe analysis on OTUs identified 13 high abundance OTUs enriched in cancer samples compared with other histological samples (Supplementary Figure 7). These OTUs can be annotated to *Flavobacterium, Klebsiella, Serratia marcescens, Stenotrophomnonas, Achromobacter* and *Pseudomonas*. Taken together, there was a robust separation between GC samples and other samples.

### Bacterial Diversity

**Figure 2:**
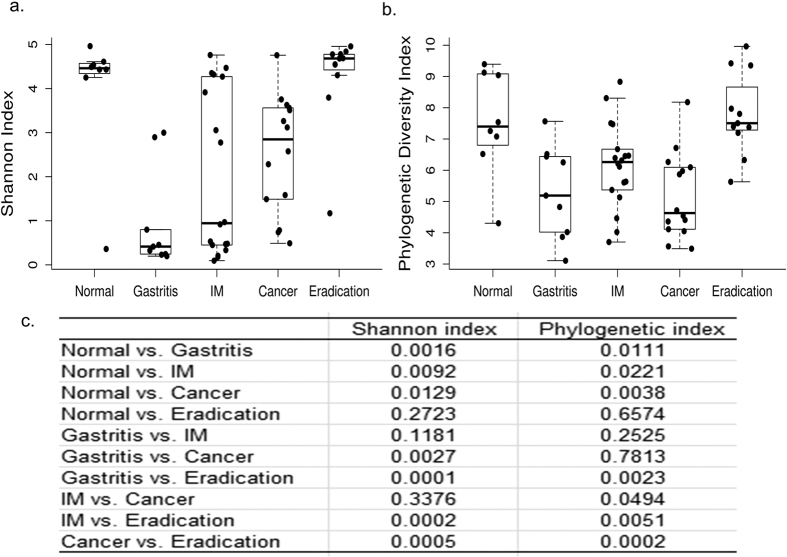
Alpha diversity in different groups. (**a**) Boxplot of Shannon index in different groups. The boxes denote interquartile ranges (IQR) with the median as a black line and whiskers extending up to the most extreme points within 1.5 fold IQR. (**b**) Boxplot of phylogenetic diversity index in different groups. The meaning of box is the same with A. (**c**) The P-values between different groups were shown. Phylogenetic index stands for phylogenetic diversity index. Mann-Whitney test was used to calculate the P-value.

Shannon entropy index and the phylogenetic diversity index were used to determine the bacterial diversity of different groups. The phylogenetic diversity index considers both phylogenetic tree distance and abundance. The normal group had the highest Shannon and phylogenetic diversity indices compared with chronic gastritis, IM and cancer groups (Fig. 2a and b), but the difference of Shannon index was larger than phylogenetic diversity index.

### Effect of *H. Pylori* Eradication on Gastric Microbiota

**Figure 3:**
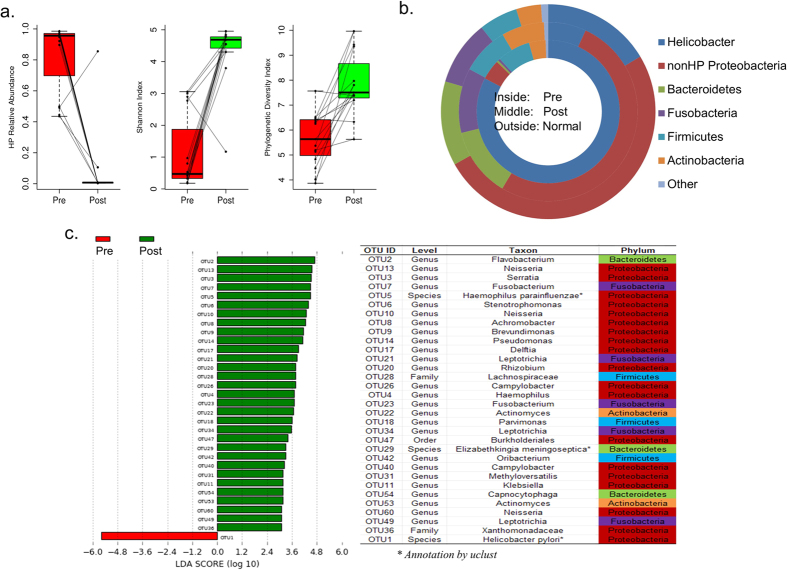
Alteration of the gastric microbiota after HP eradication. (**a**) Boxplot of the changes of HP relative abundance, Shannon index and phylogenetic diversity index before and after HP eradication. The black point indicates the sample, and the lines connected the paired samples of pre- and post-eradication. The meaning of box is the same with Fig. 2a. (**b**) Donut chart of major bacterial phyla in pre- and post-eradication samples, as well as normal control samples. (**c**) The barplot of significant OTUs with logarithmic LDA score. The OTUs were sorted according to the decreasing order of logarithmic LDA score. The taxonomic annotation of OTUs by RDP classifier was listed unless noted. The phylum column was colored according to phylum name.

Eleven HP-positive individuals with chronic gastritis or IM had serial biopsies taken before and eight weeks after receiving anti-HP treatment. The average relative abundance of *Helicobactera* decreased from 83.70% in the pre-treatment to 6.88% in the post treatment samples (Fig. 3a). Accordingly, the relative abundance of non-HP *Proteobacteria* has increased from 4.55% to 51.70%. The relative abundance of other major phyla including *Bacteroidetes, Fusobacteria* and *Actinobacteria* were also found to be increased in post-eradication samples. Both the Shannon index and phylogenetic diversity index significantly increased after HP eradication (Fig. 3a).

**Figure 4:**
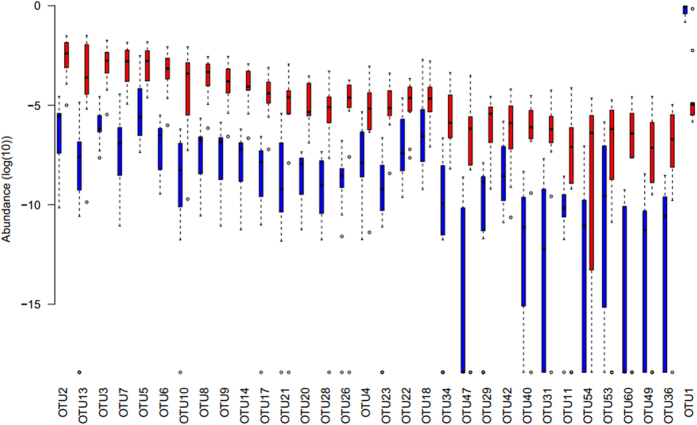
Changes in OTU abundance before and after HP eradication. The abundance distribution of OTUs were significantly different before and after HP-eradication. OTUs were showed in the same order as Fig. 3c. Blue indicates before HP-eradication group while red indicates after HP-eradication group. The meaning of box is the same with Fig. 2a.

Further analysis of the microbial composition between the post-eradication samples and the HP-negative controls showed that the relative abundance of major phyla found in these two groups were very similar (Spearman correlation coefficient = 0.93, Fig. 3b). The same pattern was observed at the genus level (Spearman correlation coefficient = 0.83). While the HP OTU was dramatically reduced in the post eradication samples, the LEfSe analysis identified 32 major OTUs that were significantly changed after HP eradication (Fig. 3c). Apart from HP that was reduced after treatment, 31 OTUs were increased after HP eradication and most of these increasing OTUs belonged to *Proteobacteria* including OTU2 (*Flavobacterium*), OTU13 (*Neisseria*), OTU3 (*Serratia*) and OTU7 (*Fusobacterium*) (Fig. 4).

## Discussion

Although the stomach is generally considered to be hostile for the growth of bacteria, there is increasing evidence to suggest that the stomach also holds a core microbiome in addition to HP^11^,^12^. With the latest Miseq sequencing platform, this study attempted to characterize the gastric microbiota in various histological stages of the gastric carcinogenesis cascade. Moreover, we have demonstrated the effects of HP eradication on changes in gastric microbiota.

First, we found that phylum *Proteobacteria* is the dominant bacterial group in the stomach, irrespective of HP infection. While HP is the dominant species in HP positive subject, non-HP *Proteobacteria* bacteria were the major players, including *Haemophilus, Serratia, Neisseria* and *Stenotrophomonas*. Even in HP-negative individuals as defined by conventional standard, some individuals still had low abundance of HP detected by the more sensitive NGS method, suggesting a false negative result by conventional biopsy based methods. The most striking difference between HP-infected and non-infected subjects was the difference in bacterial diversity, which was markedly reduced in HP-positive subjects. Moreover, the numbers of OTUs were also significantly lower in the HP-positive group, suggesting the dominance of HP infection. Previous reports that described the characteristics of gastric microbiota in HP infected subjects yielded conflicting results. Bik *et al*., by using the conventional 16 S rDNA clone library approach, found no significant difference in gastric microbiota between HP-positive and HP-negative subjects^6^. Andersson *et al*., however, showed a significant decrease in gastric microbiota diversity with HP infection by using 454 pyrosequencing technology, which was similar to our findings^12^. Despite potential difference in the distribution of HP between the antrum and corpus, we found that the corresponding gastric bacterial composition was very similar in both (Supplementary Table 3), which is in keeping with previous reports showing similar microbiota between the antrum and corpus^5^,^6^. Similar bacterial composition was also noted between tumor and adjacent non-tumorous region in patients with gastric cancer, further hinting on a common bacterial microenvironment of an individual’s stomach. We also demonstrated that age and gender of the subjects did not play a major role on bacterial composition of the stomach (Supplementary Table 3). In fact, the abundance of HP was found to have the utmost influence on the bacterial composition as well as diversity of the stomach.

Second, this is the first study to examine the serial changes in individual’s gastric microbiota before and after treatment for HP. We found that antibiotics treatment resulted in restoration of gastric microenvironment to that of HP-negative subject including similar phyla (genera) composition and an increase in the bacterial diversity index. If gastric dysbiosis plays a role on gastric carcinogenesis, eradication of HP could possibly restore the disturbed gastric homeostasis. Whilst there is increasing evidence to support the role of HP eradication in preventing gastric cancer^13^, this novel observation further supports the potential beneficial effect of HP eradication on gastric cancer prevention.

Third, we had characterized the alterations in gastric microbiota during the progression from HP gastritis to IM and gastric cancer by Illumina MiSeq platform. Whilst there was an inverse association between HP abundance and bacterial diversity in non-cancer gastric samples including normal, gastritis and IM, this inverse association was weak in cancer samples. Notably, the bacterial diversity was reduced in some cancer samples with low HP abundance. Two previous studies attempted to determine the bacterial diversity during the gastric cancer progression yielded conflicting results. Whilst one study showed progressive decline in microbial diversity during progression from gastritis to cancer^8^, the other study found increased in bacterial diversity in this progression^10^. Although both studies examined the changes during the progression, they employed different platforms for microbiota characterization including microarray chip and 454 pyrosequencing. Moreover, the study populations and sample size were also different.

By hierarchal clustering based on four different distance metrics calculated on OTU abundance, we found that the segregation of samples of different histological changes were largely attributed by the presence of HP rather than the histological stages. Nevertheless, we still observed a separation of gastric cancer samples with other samples. The usage of multiple distance metrics can overcome the bias introduced by single distance metric, revealing the robust pattern of data. Previous studies demonstrated that the IM group was distributed in between the chronic gastritis and gastric cancer group^8^,^10^. Both studies showed that IM was found to overlap in the gastritis and cancer group by hierarchical clustering based on Weighted Unifrac distance and UPGMA clustering among patients with HP dominance. Our results showed that gastritis samples were largely overlapped with IM samples. The difference may lie on the recruitment of HP-positive subjects only in previous studies^8^,^10^. The use of a more robust sequencing platform in our study, which yielded more OTUs, may also account for the discrepancies.

There are increasing evidences based on animal studies to suggest a possible link between alteration of gastric microbiota and gastric cancer development. In germ-free mice studies, it was shown that the lack of commensal flora delays the development of intraepithelial neoplasia, suggesting the importance of gastric microbiota in the promotion of gastric carcinogenesis in achlorhydric stomach^14^. The presence of intestinal flora was also found to enhance the inflammatory responses in the stomach in HP infected mice, which may promote the development of gastric atrophy and neoplasia development^15^. Results from human study further suggested that the lower microbial richness in upper digestive tract was associated with lower pepsinogen I/II ratio, which is a gastric cancer predisposing state^9^. The findings of this study may open up another area of research on the potential reversibility of gastric microbiome by treatment of HP.

This study however had limitations. First, whilst it is believed that the gastric microbiota is dynamic and can be influenced by many external factors such as drug or diet^16^, we have excluded subjects with recent intake of proton pump inhibitor (PPI), non-steroidal anti-inflammatory drug (NSAID) or aspirin. As all biopsies were taken at fasting state during endoscopy, the potential influence by meal was kept to a minimum. Although we have taken serial biopsies from patients with HP infection, we have not taken serial biopsies from those without HP infection to determine the stability of gastric microbiota over time. However, there are ethical issues in repeating biopsy in those with normal gastric histology and no HP infection. Second, data form this study that based on next generation sequencing could only produce the distribution or relative abundance of different bacteria in the stomach. Actual quantitative determination of individual bacteria in different samples is not possible and further quantitative techniques such as PCR are needed for this specific purpose.

In conclusion, we showed that HP dominated the gastric microbiota in those with HP infection resulting in reduced bacterial diversity and alteration in the relative abundance of other bacterial species. Eradication of HP restored the bacterial diversity and composition of bacteria to levels similar to HP negative subjects. During progression from HP gastritis to IM and cancer, cancer samples had reduction in bacterial diversity and enhancement of certain bacterial species. Further studies are needed to determine whether modulation of gastric microbiota could potentially alter the risk of progression to gastric cancer in HP infected individuals.

## Methods

### Patients and samples

Study subjects were enrolled from adult patients who underwent upper gastrointestinal endoscopy in the Queen Mary Hospital of Hong Kong. We recruited patients with HP associated gastritis and different gastric histological changes including gastric IM and GC. Age-matched HP-negative subjects were recruited as negative control. Patients who were found to have active gastroduodenal ulcers on endoscopy were excluded. We also excluded patients who were taking acid suppressive therapy including PPIs and histamine (H2)-receptor blocker, NSAIDs, anti-coagulants, anti-platelet medications or had taken antibiotics within recent four weeks. Individuals who had previously undergone upper gastrointestinal endoscopy, prior gastric surgery or had received HP eradication treatment were excluded.

During upper gastrointestinal endoscopy, gastric biopsies were obtained from the antrum and corpus of the stomach in patients without GC for rapid urease test, histological examination and DNA extraction. *H. pylori* infection was defined by a positive rapid urease test and/or visualization of the bacteria on histological examination. Targeted biopsies were taken from those with endoscopic evidence of IM. Diagnosis of IM and GC were subsequently verified by histological examination. In patients with GC, biopsies were taken from the tumor and adjacent non-cancerous area. Gastric biopsy samples were either stored in 10% buffered formalin for histological examination or immediately immerged in RNALater^®^ solution (Life Technologies, Carlsbad, USA) and then stored at −80 °C for subsequent DNA extraction.

All HP infected patients were given a one-week course of eradication therapy consisting of esomeprazole 20 mg, amoxicillin 1 g and clarithromycin 500 mg, all given twice daily. Patients who were allergic to penicillin were given metronidazole 400 mg twice daily instead of amoxicillin. Eight weeks after completion of the HP eradication treatment, they were invited for repeat endoscopy and gastric biopsy.

### Consent and Institutional Review Board

All patients provided written informed consent for participation in this study. The study protocol was approved by the Institutional Review Board of the University of Hong Kong and the Hospital Authority-Hong Kong West Cluster (UW 11-354). The study was performed in accordance with the Declaration of Helsinki.

### DNA Extraction, Purification and Solexa Illumina Sequencing

Total DNA was extracted from the gastric biopsies using the QIAGEN DNeasy Kit (QIAGEN, Venlo, Netherlands). Sequencing works were performed by the Illumina MiSeq sequencer (Illumina, San Diego, USA). The KAPA HiFi HotStart ReadMix PCR Kit (Kapa Biosystems) and Nextera XT Index Kit FCD-131-1001 (Illumina) were used for PCR. These libraries were then cleaned up and quantified using the KAPA Library Quantification Kit KK4835 for Illumina sequencing platforms (Kapa Biosystems). The MiSeq Reagent Kits v3 MS-102-3003 (Illumina) together with PhiX Control v3 (Illumina) were used for sequencing.

### Sequencing Data Processing

Trimmomatic^17^ was used to filter low quality reads and adapter sequences with parameters MAXINFO: 40:0.38, which balanced the quality and reads length. PEAR^18^ was then used to merge the paired-end reads with parameters -t 170 -q 20 -u 0. Primer sequences attached to merge reads were identified by BLAT^19^ alignment and trimmed. Subsequently, these reads were processed with the UPARSE program^20^ to detect chimera sequences and clustered into operational taxonomic units (OTUs) at 97% sequence similarity.

For taxonomic assignment, the most abundant sequences were chosen as the representative sequences of corresponding OTUs. The taxonomic annotation of representative sequences was deemed as the taxonomic annotation of OTUs. To obtain robust taxonomic annotation, both RDP classifier^21^ and UCLUST (version 1.2.22)^22^ methods implemented in QIIME^23^ were used. The reference database used was Green gene database (release 13_8)^24^. The default parameters (the confidence cutoff of RDP is 0.8, and the identity cutoff of UCLUST is 97%) were used for this step.

The rarefaction analysis was performed using QIIME to evaluate the effect of sequencing depth. The sampling started from 88 sequences per sample, with the step of 400 sequences, ended at 10,088 sequences per sample. Each step was repeated 1,000 times. Observed species number and Chao 1 index were calculated. To reduce the effects of various sequencing depth, the sequencing data were subsampled to 10,088 reads (the minimum sampling size) to calculate the Shannon index and phylogenetic diversity^25^ index. Similarly, the unweighted and weighted unifrac distances were also calculated based on the subsampled dataset. Another two distance metrics, the root Jensen-Shannon divergence (rJSD)^26^ and Bray-Curtis dissimilarity were calculated based on the normalized OTUs relative abundance table. Principle coordinate analysis (PCoA) was performed on rJSD distance matrix. Hierarchical clustering was performed on these four distance matrixes with *ward* method. The same analysis was conducted after removing HP OTUs.

### Statistical Analysis

PERMANOVA^27^ was implemented to evaluate the features associated with the sequencing data. These features included age, gender, HP state, sampling location, and HP-eradication. LEfSe^28^ was used to identify the biomarkers significantly enriched in different histological group. Wilcoxon rank-sum test (Mann-Whitney test) was performed to infer the significance difference of diversity and microbial compositions between different histological states. Paired Wilcoxon rank sum test was used to identify the significant changes of taxa in pre- and post-eradication samples. Spearman correlation coefficient was used to evaluate the degree of association.

## Additional Information

**How to cite this article:** Li, TH. *et al*. Alterations in Gastric Microbiota After *H. Pylori* Eradication and in Different Histological Stages of Gastric Carcinogenesis. *Sci. Rep.* **7**, 44935; doi: 10.1038/srep44935 (2017).

**Publisher's note:** Springer Nature remains neutral with regard to jurisdictional claims in published maps and institutional affiliations.

## Supplementary Material

Supplementary Tables and Figures
