# Supplementary material for: Alterations in Gastric Microbiota After H. Pylori Eradication and in Different Histological Stages of Gastric Carcinogenesis
Source: Sci Rep. 2017 Mar 21;7:44935. doi: 10.1038/srep44935 (PMC5359573; doi:10.1038/srep44935)
Supplement: Supplementary Tables and Figures [file srep44935-s1.pdf]

SUPPLEMENTARY TABLES AND FIGURES

ALTERATIONS IN GASTRIC MICROBIOTA AFTER *H. PYLORI* ERADICATION AND IN DIFFERENT HISTOLOGICAL STAGES OF GASTRIC CARCINOGENESIS

Tung Hiu Li[1], Youwen Qin[2], Pak Chung Sham[2], K.S. Lau[1], Kent-Man Chu[3], Wai K. Leung[1*]

[1]Department of Medicine, The University of Hong Kong, Hong Kong

[2]Centre for Genomic Sciences, The University of Hong Kong, Hong Kong

[3]Department of Surgery, The University of Hong Kong, Hong Kong

Supplementary Table 1: Patient's samples

Study_ID	Gender	Age	HP_Positive	Disease	OTUs_Count	Shannon_Index
Normal1	F	48	No	Normal	172	4.9633
Normal2	F	54	No	Normal	193	4.5347
Normal3	M	63	No	Normal	130	4.4301
Normal4	F	57	No	Normal	160	4.6102
Normal5	M	40	No	Normal	109	0.3601
Normal6	F	57	No	Normal	100	4.2514
Normal7	F	32	No	Normal	128	4.432
Normal8	M	42	No	Normal	131	4.4909
Gastritis1	M	59	Yes	Gastritis	191	2.9994
Gastritis2	F	33	Yes	Gastritis	88	0.1977
Gastritis3	F	47	Yes	Gastritis	166	0.454
Gastritis4	F	52	Yes	Gastritis	209	2.8965
Gastritis5	F	56	Yes	Gastritis	87	0.2445
Gastritis6	F	54	Yes	Gastritis	128	0.3252
Gastritis7	F	58	Yes	Gastritis	228	0.799
Gastritis8	M	48	Yes	Gastritis	139	0.4143
Gastritis9	F	25	Yes	Gastritis	72	0.2304
IM1_1	M	48	No	IM	178	4.7613
IM1_2	M	48	No	IM	126	4.3518
IM2_1	F	61	Yes	IM	210	2.7762
IM2_2	F	61	Yes	IM	90	0.1785
IM3_1	F	36	Yes	IM	166	0.4707
IM3_2	F	36	Yes	IM	181	3.0559
IM4_1	F	56	No	IM	119	4.3199
IM4_2	F	56	No	IM	104	3.9152
IM5_1	M	70	Yes	IM	160	0.969
IM5_2	M	70	Yes	IM	148	0.5309
IM6_1	M	49	Yes	IM	153	0.9198
IM6_2	M	49	Yes	IM	78	0.0945
IM7_1	M	49	No	IM	165	4.4695
IM7_2	M	49	No	IM	130	4.2714
IM8_1	F	56	Yes	IM	139	0.2054
IM8_2	F	56	Yes	IM	141	0.3339
IM9_1	F	54	Yes	IM	135	0.4793
IM9_2	F	54	Yes	IM	164	0.4498
Cancer1_1	M	70	No	Cancer	119	3.2632
Cancer1_2	M	70	No	Cancer	104	3.7544
Cancer2_1	M	48	No	Cancer	85	3.629
Cancer2_2	M	48	No	Cancer	59	3.5095
Cancer3_1	F	61	Yes	Cancer	78	0.7841
Cancer3_2	F	61	Yes	Cancer	80	2.2833
Cancer4_1	M	56	No	Cancer	57	3.1189
Cancer4_2	M	56	No	Cancer	109	2.5765
Cancer5_1	F	49	No	Cancer	76	1.4901
Cancer5_2	F	49	No	Cancer	64	0.4897
Cancer6_1	M	36	Yes	Cancer	84	1.5837
Cancer6_2	M	36	Yes	Cancer	152	0.7406
Cancer7_1	M	54	No	Cancer	168	4.7575
Cancer7_2	M	54	No	Cancer	96	3.5625
Gastritis1_E	M	59	No	Eradication	159	4.5446
Gastritis2_E	F	33	No	Eradication	189	4.5516
Gastritis3_E	F	47	No	Eradication	188	4.9577
Gastritis4_E	F	52	Yes	Eradication	169	1.1708
IM2_E	F	61	No	Eradication	154	4.3028
IM3_E	F	36	No	Eradication	147	4.6894
Gastritis6_E	F	54	No	Eradication	153	4.6833
IM5_E	M	70	No	Eradication	202	4.7769
Gastritis7_E	F	58	No	Eradication	146	4.7808
Gastritis8_E	M	48	No	Eradication	90	3.7961
IM8_E	F	56	No	Eradication	145	4.8402

HP, *Helicobacter pylori*; IM, intestinal metaplasia; OTU, operational taxonomic unit

Supplementary Table 2: Processing of sequencing reads

	Removing adapters, Filtering and Trimming low quality reads							erging paired-end re		ers filtering and trim		Filtering chimeras		
Experiment_ID	# reads (paired)	# paired surviving	paired survivi	ard only sur	ard only s	rse only su	rse only su	merged re	%	rimmed re	%	-chemira	%	Final surviving
wk1043	265,975	166,735	62.69	55,317	20.80	6,548	2.46	122,789	73.64	89,973	73.27	88,714	98.60	33.35
wk1053	295,704	230,792	78.05	34,615	11.71	7,364	2.49	203,751	88.28	187,853	92.20	186,482	99.27	63.06
wk1061	235,106	165,449	70.37	39,298	16.72	5,157	2.19	141,930	85.79	129,184	91.02	128,410	99.40	54.62
wk1062	171,961	121,334	70.56	29,143	16.95	3,433	2.00	106,399	87.69	99,626	93.63	99,025	99.40	57.59
wk1123	140,648	90,543	64.38	27,199	19.34	3,667	2.61	66,624	73.58	49,980	75.02	49,523	99.09	35.21
wk1154	278,047	185,553	66.73	54,560	19.62	5,577	2.01	135,689	73.13	125,743	92.67	125,347	99.69	45.08
wk1155	207,590	142,435	68.61	35,728	17.21	4,875	2.35	101,236	71.08	94,913	93.75	94,598	99.67	45.57
wk1163	235,130	177,487	75.48	31,574	13.43	5,378	2.29	155,541	87.64	144,657	93.00	137,706	95.19	58.57
wk1201	229,972	134,103	58.31	53,936	23.45	4,743	2.06	95,290	71.06	75,597	79.33	75,097	99.34	32.65
wk1202	165,285	90,569	54.80	46,330	28.03	2,472	1.50	66,726	73.67	55,456	83.11	55,182	99.51	33.39
wk1213	240,516	148,961	61.93	49,496	20.58	6,618	2.75	104,514	70.16	75,202	71.95	73,938	98.32	30.74
wk1221	314,709	240,825	76.52	42,140	13.39	7,284	2.31	214,454	89.05	200,074	93.29	199,545	99.74	63.41
wk1222	306,554	226,231	73.80	47,441	15.48	6,440	2.10	199,634	88.24	184,615	92.48	184,067	99.70	60.04
wk1241	244,063	179,898	73.71	36,669	15.02	5,224	2.14	158,435	88.07	148,270	93.58	141,214	95.24	57.86
wk1242	257,396	193,146	75.04	36,080	14.02	5,766	2.24	170,078	88.06	158,568	93.23	151,114	95.30	58.71
wk1263	119,201	66,496	55.78	31,896	26.76	1,807	1.52	49,235	74.04	41,898	85.10	41,724	99.58	35.00
wk1264	270,250	193,976	71.78	44,295	16.39	6,068	2.25	164,491	84.80	152,270	92.57	151,697	99.62	56.13
wk1265	252,737	186,468	73.78	34,451	13.63	6,969	2.76	159,412	85.49	148,463	93.13	147,814	99.56	58.49
wk1293	220,101	125,010	56.80	53,469	24.29	4,307	1.96	87,451	69.96	40,847	46.71	40,378	98.85	18.35
wk1303	233,519	130,204	55.76	64,676	27.70	3,292	1.41	98,040	75.30	78,911	80.49	78,558	99.55	33.64
wk1313	263,741	189,811	71.97	42,620	16.16	5,418	2.05	165,804	87.35	155,474	93.77	155,138	99.78	58.82
wk1394	237,449	149,840	63.10	46,426	19.55	6,778	2.85	107,376	71.66	77,541	72.21	77,205	99.57	32.51
wk1395	145,422	86,734	59.64	36,628	25.19	2,544	1.75	65,759	75.82	56,889	86.51	56,587	99.47	38.91
wk204	156,261	93,719	59.98	38,607	24.71	3,027	1.94	70,718	75.46	62,216	87.98	62,015	99.68	39.69
wk205	51,486	34,711	67.42	9,147	17.77	1,418	2.75	26,249	75.62	22,980	87.55	22,865	99.50	44.41
wk213	200,018	130,488	65.24	24,067	12.03	14,397	7.20	79,029	60.56	62,283	78.81	61,965	99.49	30.98
wk263	212,761	132,416	62.24	29,294	13.77	10,653	5.01	93,842	70.87	88,056	93.83	79,099	89.83	37.18
wk303	210,818	159,146	75.49	26,773	12.70	6,120	2.90	136,635	85.86	127,910	93.61	127,567	99.73	60.51
wk354	324,118	232,361	71.69	55,563	17.14	6,107	1.88	203,620	87.63	189,859	93.24	189,333	99.72	58.41
wk355	207,443	144,658	69.73	37,186	17.93	4,709	2.27	117,536	81.25	107,447	91.42	107,085	99.66	51.62
wk423	180,281	120,002	66.56	16,329	9.06	18,615	10.33	69,064	57.55	51,759	74.94	50,909	98.36	28.24
wk424	109,671	20,910	19.07	65,013	59.28	853	0.78	12,031	57.54	10,138	84.27	10,088	99.51	9.20
wk425	203,211	132,609	65.26	42,227	20.78	4,184	2.06	92,013	69.39	60,569	65.83	58,542	96.65	28.81
wk433	234,335	161,117	68.75	27,942	11.92	14,168	6.05	100,988	62.68	80,709	79.92	80,294	99.49	34.26
wk441	304,561	194,648	63.91	57,672	18.94	8,270	2.72	147,470	75.76	115,172	78.10	113,015	98.13	37.11
wk442	199,078	126,633	63.61	42,292	21.24	4,244	2.13	98,942	78.13	85,574	86.49	84,504	98.75	42.45
wk473	201,106	132,523	65.90	24,423	12.14	13,697	6.81	84,146	63.50	65,085	77.35	64,171	98.60	31.91
wk493	239,339	180,959	75.61	20,970	8.76	14,048	5.87	145,620	80.47	136,777	93.93	136,348	99.69	56.97
wk523	248,445	185,322	74.59	31,340	12.61	7,666	3.09	155,725	84.03	145,118	93.19	144,421	99.52	58.13
wk563	280,080	179,471	64.08	39,641	14.15	7,417	2.65	149,874	83.51	140,957	94.05	128,928	91.47	46.03
wk583	110,260	71,716	65.04	21,709	19.69	2,749	2.49	53,443	74.52	47,251	88.41	46,979	99.42	42.61
wk611	280,001	167,124	59.69	57,521	20.54	5,167	1.85	140,947	84.34	131,970	93.63	121,693	92.21	43.46
wk612	210,104	139,263	66.28	44,342	21.10	4,329	2.06	117,481	84.36	109,522	93.23	108,404	98.98	51.60
wk643	212,512	142,044	66.84	36,619	17.23	6,100	2.87	104,132	73.31	78,083	74.98	77,436	99.17	36.44
wk671	295,579	224,535	75.96	38,758	13.11	7,504	2.54	197,228	87.84	185,163	93.88	183,909	99.32	62.22
wk672	201,826	117,308	58.12	36,547	18.11	5,158	2.56	92,927	79.22	87,099	93.73	77,434	88.90	38.37
wk723	208,815	159,892	76.57	26,341	12.61	5,045	2.42	140,299	87.75	131,112	93.45	130,795	99.76	62.64
wk74	259,193	156,259	60.29	63,654	24.56	5,134	1.98	112,907	72.26	100,665	89.16	100,252	99.59	38.68
wk75	219,938	142,702	64.88	43,404	19.73	5,422	2.47	105,051	73.62	92,080	87.65	91,489	99.36	41.60
wk763	201,277	132,937	66.05	34,722	17.25	6,048	3.00	98,129	73.82	71,940	73.31	71,392	99.24	35.47
wk813	193,677	126,740	65.44	37,997	19.62	4,451	2.30	95,512	75.36	69,191	72.44	68,465	98.95	35.35
wk833	215,462	161,167	74.80	33,506	15.55	3,670	1.70	144,615	89.73	135,309	93.56	134,809	99.63	62.57
wk863	270,245	204,272	75.59	37,650	13.93	5,760	2.13	176,678	86.49	160,948	91.10	159,514	99.11	59.03
wk893	253,279	175,473	69.28	37,928	14.97	9,022	3.56	123,375	70.31	89,277	72.36	88,407	99.03	34.90
wk903	224,345	148,120	66.02	41,877	18.67	6,026	2.69	111,008	74.95	92,517	83.34	91,824	99.25	40.93
wk931	287,905	179,160	62.23	60,928	21.16	7,113	2.47	133,690	74.62	106,646	79.77	106,291	99.67	36.92
wk932	136,049	82,767	60.84	32,957	24.22	2,514	1.85	63,340	76.53	54,707	86.37	54,439	99.51	40.01
wk961	333,142	253,698	76.15	42,696	12.82	8,871	2.66	219,670	86.59	203,660	92.71	202,087	99.23	60.66
wk962	258,623	190,902	73.81	38,993	15.08	5,622	2.17	166,263	87.09	154,668	93.03	153,281	99.10	59.27
wk993	309,450	203,633	65.80	52,752	17.05	9,623	3.11	148,169	72.76	100,022	67.51	99,580	99.56	32.18
Total	13,575,770	9,164,005	-	2,383,374	-	372,650	-	7,269,024	-	6,326,443	-	6,228,692	-	-
Average	226,263	152,733	66.47	39,723	18.29	6,211	2.76	121,150	77.81	105,441	85.50	103,812	98.50	44.54
sd	56,992	47,797	8.74	11,894	6.98	3,316	1.54	45,801	8.33	46,271	9.65	45,764	2.37	12.65

Supplementary Table 3: The PERMANOVA results of different group comparisons

Feature	Jensena_Shannon	Bray_Curtis	Weighted_unifrac	Unweighted_unifrac
Gender	0.0658	0.0896	0.1256	0.2082
Age	0.7229	0.7957	0.8236	0.9414
Helicobacter_pylori_state	0.0005	0.0008	0.0008	0.0019
Disease (Normal vs. Gastritis vs. IM vs. Cancer)	0.0005	0.0011	0.0012	0.0008
Antrum vs. Corpus	0.9817	0.9794	0.9366	0.9414
Tumor vs. Adjacent tissue	0.9817	0.9794	0.9366	0.9414
NAD vs. Gastritis	0.0012	0.0012	0.0012	0.0141
NAD vs. IM	0.0377	0.0360	0.0437	0.1700
NAD vs. Cancer	0.3468	0.5004	0.4420	0.0087
Gastritis vs. IM	0.1212	0.1224	0.1296	0.5041
Gastritis vs. Cancer	0.0035	0.0029	0.0067	0.0050
IM vs.Cancer	0.0462	0.0866	0.1296	0.0011

Supplementary Figure 1: Rarefaction curves

A: Rarefaction curves of observed species number;

B: Rarefaction curves of Chao 1 index.

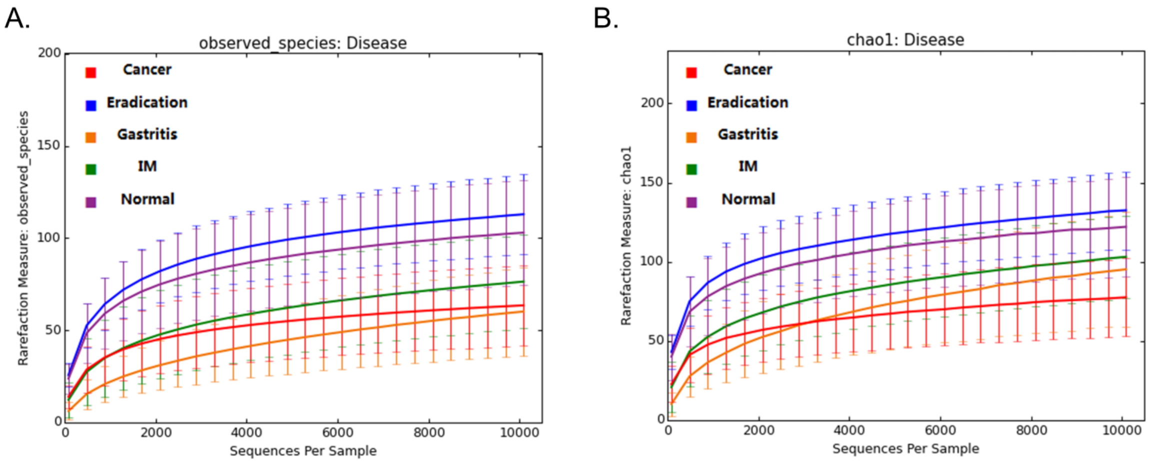

Each line represents one histological group. Colors indicate different groups. The sampling started from 88 sequences per sample, with step of 400 sequences, ended at 10,088 sequences per sample. Every step was repeated 1,000 times.

Supplementary Figure 2: Bar-plot of major phyla and major genera.

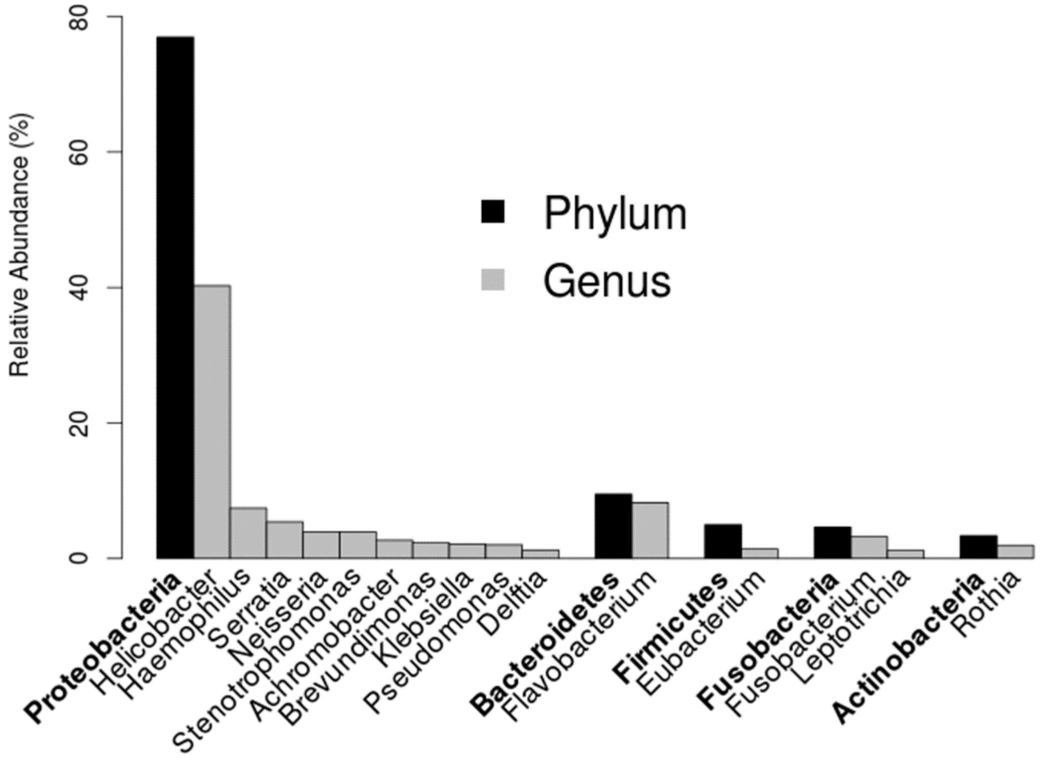

The phyla and genera were sorted by the decreasing order of relative abundance. Only taxa with relative abundance >1% were showed.

Supplementary Figure 3: Relative abundance distribution of major phyla across 60 samples.

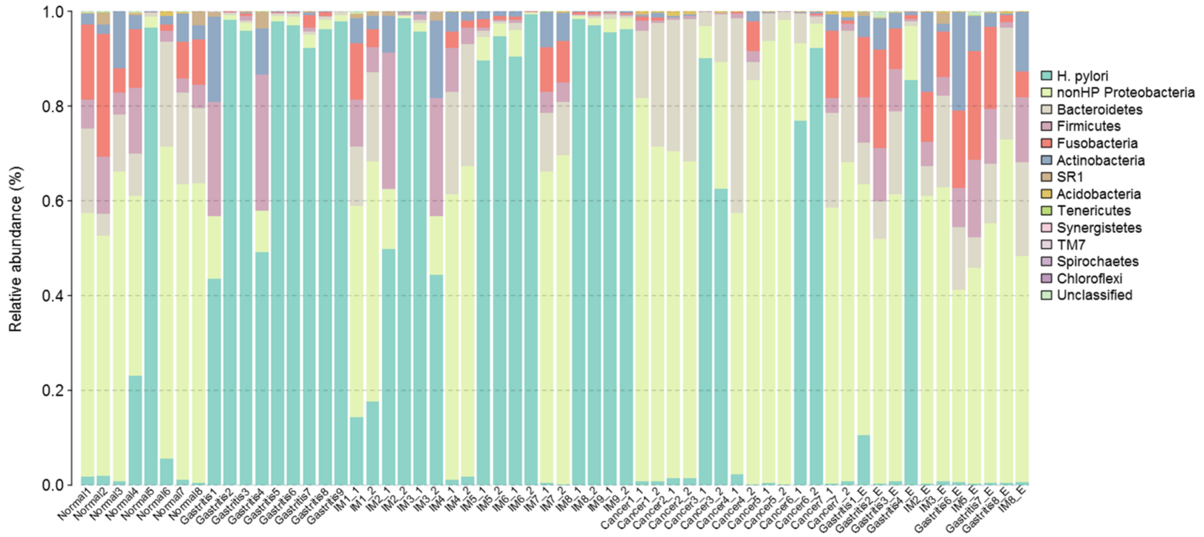

The phyla were sorted according to the decreasing order of the average relative abundance. All detected phyla were showed and *Proteobacteria* was divided into *H. pylori* and non-HP *Proteobacteria*. Genera were sorted by the decreasing order of average relative abundance. Samples were ordered by their histological information. Sample names have the same meaning with Figure 1A.

Supplementary Figure 4: Principle coordinate analysis (PCoA) based on OTUs relative abundance profile without HP OTUs.

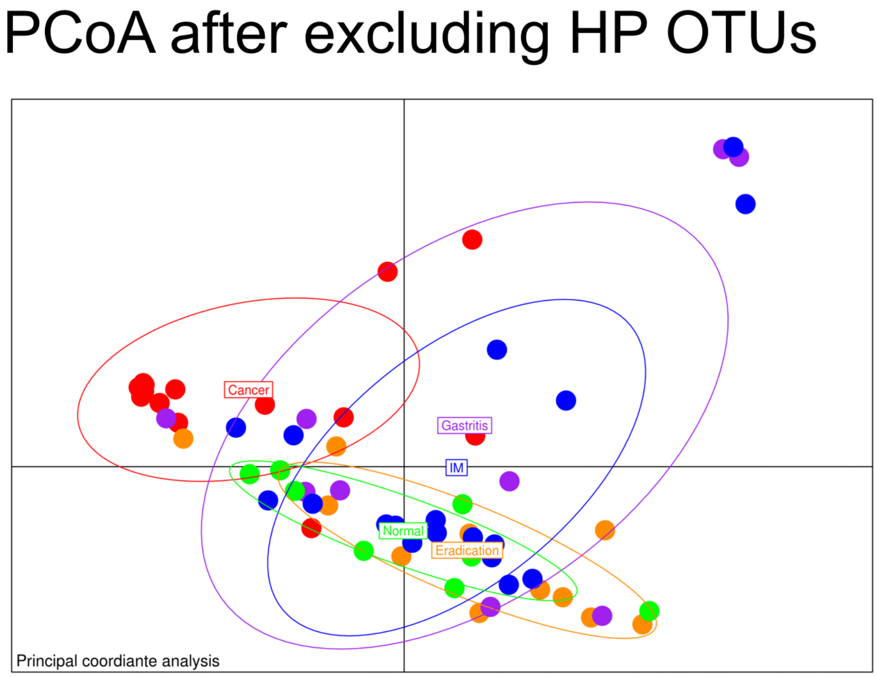

The variance explained by PC1 and PC2 are 30.6%% and 15.3%, respectively. The distance was measured by root Jessen-Shannon divergence (rJSD). Points represent samples, and the color indicates histological group.

Supplementary Figure 5: The dendrograms of hierarchical clustering results.

A. Rooted Jensen-Shannon divergence distance;

B. Bray-Curtis dissimilarity;

C. Weighted unifrac distance;

D. Unweighted unifrac distance

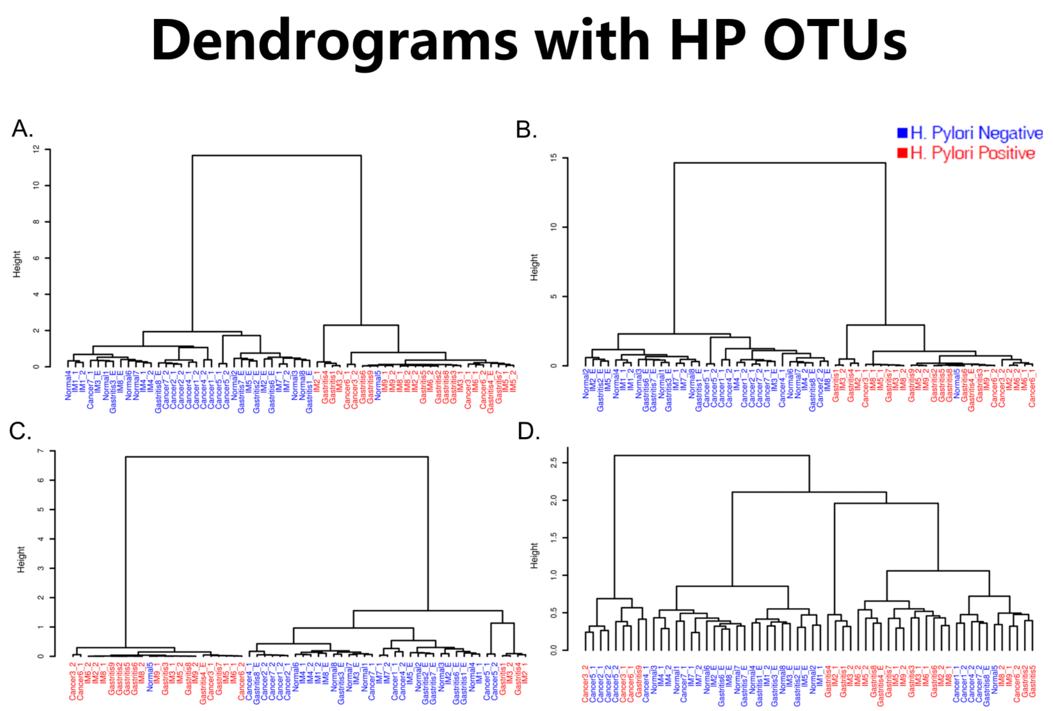

The clustering method for hierarchical clustering was *ward*. Sample names indicates their histological information and colored by HP state, red—HP positive, blue—HP negative.

Supplementary Figure 6: The dendrograms of hierarchical clustering results (excluding HP OTUs).

A. Rooted Jensen-Shannon divergence distance;

B. Bray-Curtis dissimilarity;

C. Weighted unifrac distance;

D. Unweighted unifrac distance;

Dendrograms without HP OTUs

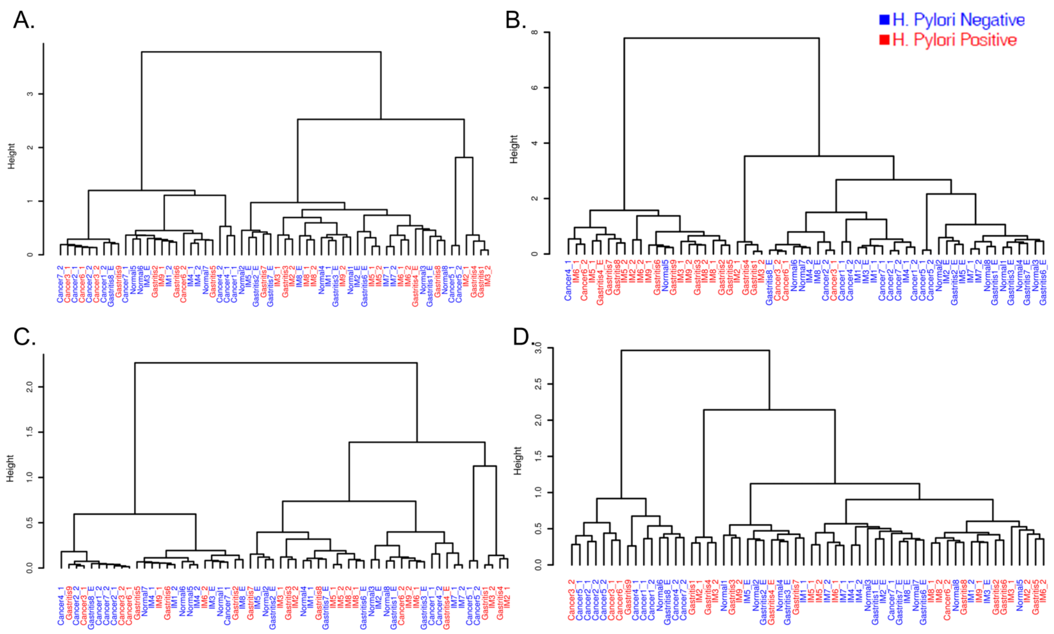

The clustering method for hierarchical clustering was *ward*. Sample names indicate their histological information and colored by HP state, red—HP positive, blue—HP negative.

Supplementary figure 7: The OTUs significantly enriched in different histological groups.

G1: Normal, G2: HP Gastritis, G3: Gastric IM, G4: Gastric Cancer.

OTUs were sorted by enriched groups and decreasing order of logarithmic LDA score. The right shows the taxonomic results of RDP classifier unless noted. The phylum column was colored according to the phylum name. Only the top 96 high abundance OTUs with LDA score >3.0 were shown.

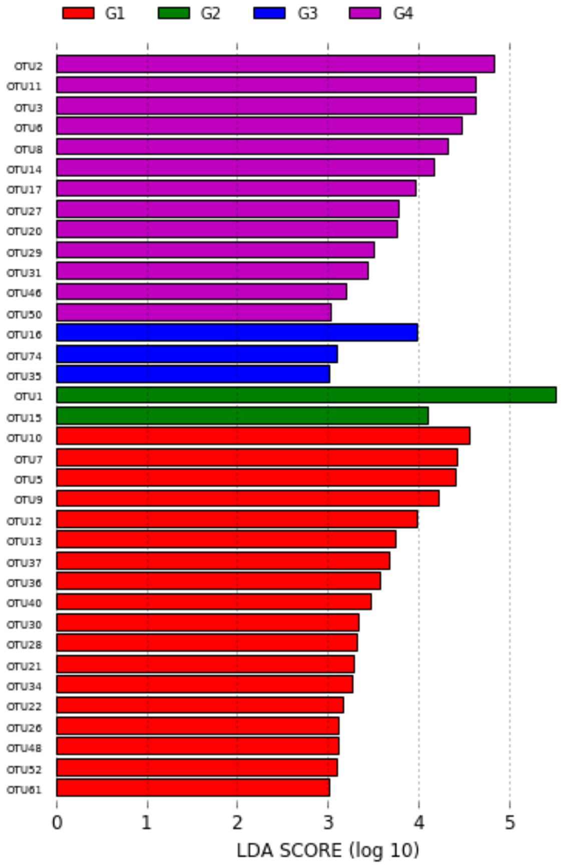

OTU ID	Level	Taxon	Phylum
OTU2	Genus	Flavobacterium	Bacteroidetes
OTU11	Genus	Klebsiella	Proteobacteria
OTU3	Species	Serratia marcescens*	Proteobacteria
OTU6	Genus	Stenotrophomonas	Proteobacteria
OTU8	Genus	Achromobacter	Proteobacteria
OTU14	Genus	Pseudomonas	Proteobacteria
OTU17	Genus	Delftia	Proteobacteria
OTU27	Genus	Ralstonia	Proteobacteria
OTU20	Genus	Rhizobium	Proteobacteria
OTU29	Species	Elizabethkingia meningoseptica*	Bacteroidetes
OTU31	Genus	Methyloversatilis	Proteobacteria
OTU46	Order	Gp4	Acidobacteria
OTU50	Family	Cytophagaceae*	Bacteroidetes
OTU16	Species	Actinobacillus parahaemolyticus*	Proteobacteria
OTU74	Genus	Fusobacterium	Fusobacteria
OTU35	Genus	Leptotrichia	Fusobacteria
OTU1	Species	Helicobacter pylori*	Proteobacteria
OTU15	Genus	Eubacterium	Firmicutes
OTU10	Genus	Neisseria	Proteobacteria
OTU7	Genus	Fusobacterium	Fusobacteria
OTU5	Species	Haemophilus parainfluenzae*	Proteobacteria
OTU9	Genus	Brevundimonas	Proteobacteria
OTU12	Species	Rothia mucilaginosa*	Actinobacteria
OTU13	Genus	Neisseria	Proteobacteria
OTU37	Species	Aggregatibacter segnis*	Proteobacteria
OTU36	Family	Xanthomonadaceae	Proteobacteria
OTU40	Genus	Campylobacter	Proteobacteria
OTU30	Genus	Peptostreptococcus	Firmicutes
OTU28	Family	Lachnospiraceae	Firmicutes
OTU21	Genus	Leptotrichia	Fusobacteria
OTU34	Genus	Leptotrichia	Fusobacteria
OTU22	Genus	Actinomyces	Actinobacteria
OTU26	Genus	Campylobacter	Proteobacteria
OTU48	Genus	Eubacterium	Firmicutes
OTU52	Order	Burkholderiales	Proteobacteria
OTU61	Speceis	Porphyromonas endodontalis*	Bacteroidetes

* *Annotation by uclust*
